# Effectiveness of Web-Based Personalized Nutrition Advice for Adults Using the eNutri Web App: Evidence From the EatWellUK Randomized Controlled Trial

**DOI:** 10.2196/29088

**Published:** 2022-04-25

**Authors:** Rodrigo Zenun Franco, Rosalind Fallaize, Michelle Weech, Faustina Hwang, Julie A Lovegrove

**Affiliations:** 1 Biomedical Engineering School of Biological Sciences University of Reading Reading United Kingdom; 2 Hugh Sinclair Unit of Human Nutrition and Institute for Cardiovascular and Metabolic Research University of Reading Reading United Kingdom; 3 School of Life and Medical Sciences University of Hertfordshire Hatfield United Kingdom

**Keywords:** personalized nutrition, web-based, nutrition app, app, dietary intervention, eNutri, precision nutrition, mHealth, healthy eating index, diet quality scores, FFQ, food frequency questionnaire, EatWellUK

## Abstract

**Background:**

Evidence suggests that eating behaviors and adherence to dietary guidelines can be improved using nutrition-related apps. Apps delivering personalized nutrition (PN) advice to users can provide individual support at scale with relatively low cost.

**Objective:**

This study aims to investigate the effectiveness of a mobile web app (eNutri) that delivers automated PN advice for improving diet quality, relative to general population food-based dietary guidelines.

**Methods:**

Nondiseased UK adults (aged >18 years) were randomized to PN advice or control advice (population-based healthy eating guidelines) in a 12-week controlled, parallel, single-blinded dietary intervention, which was delivered on the web. Dietary intake was assessed using the eNutri Food Frequency Questionnaire (FFQ). An 11-item US modified Alternative Healthy Eating Index (m-AHEI), which aligned with UK dietary and nutritional recommendations, was used to derive the automated PN advice. The primary outcome was a change in diet quality (m-AHEI) at 12 weeks. Participant surveys evaluated the PN report (week 12) and longer-term impact of the PN advice (mean 5.9, SD 0.65 months, after completion of the study).

**Results:**

Following the baseline FFQ, 210 participants completed at least 1 additional FFQ, and 23 outliers were excluded for unfeasible dietary intakes. The mean interval between FFQs was 10.8 weeks. A total of 96 participants were included in the PN group (mean age 43.5, SD 15.9 years; mean BMI 24.8, SD 4.4 kg/m^2^) and 91 in the control group (mean age 42.8, SD 14.0 years; mean BMI 24.2, SD 4.4 kg/m^2^). Compared with that in the control group, the overall m-AHEI score increased by 3.5 out of 100 (95% CI 1.19-5.78) in the PN group, which was equivalent to an increase of 6.1% (*P*=.003). Specifically, the m-AHEI components *nuts and legumes* and *red and processed meat* showed significant improvements in the PN group (*P*=.04). At follow-up, 64% (27/42) of PN participants agreed that, compared with baseline, they were still following some (*any*) of the advice received and 31% (13/42) were still motivated to improve their diet.

**Conclusions:**

These findings suggest that the eNutri app is an effective web-based tool for the automated delivery of PN advice. Furthermore, eNutri was demonstrated to improve short-term diet quality and increase engagement in healthy eating behaviors in UK adults, as compared with population-based healthy eating guidelines. This work represents an important landmark in the field of automatically delivered web-based personalized dietary interventions.

**Trial Registration:**

ClinicalTrials.gov NCT03250858; https://clinicaltrials.gov/ct2/show/NCT03250858

## Introduction

### Background

Noncommunicable diseases account for almost two-thirds of deaths globally [1]. The main recommendations for addressing noncommunicable diseases are related to lifestyle changes, such as the encouragement of healthier diets, physical activity (PA), and the reduction of tobacco use and alcohol consumption [1]. Current public health strategies that aim to address this challenge, for example, the United Kingdom’s *5-a-day* campaign that encourages the consumption of at least 5 portions of a variety of fruits and vegetables daily [2] and the Eatwell Guide [3], are not personalized to individuals. Although the *5-a-day* campaign was associated with modest increases, particularly in fruit consumption, immediately after its launch [4], these were not maintained and currently only a third of UK adults meet the recommendations of this campaign [5]. These and other data have motivated investigations into the efficacy of personalized nutrition (PN) advice on dietary behavior change [6]. The promise of PN may lie in having a greater capacity to motivate individuals to change their dietary habits or the delivery of more suitable and thus more effective advice. For example, tailored health information is perceived as more personally relevant [7], and often superior [8], by consumers, and has also been shown to stimulate greater cognitive activity (eg, being read and remembered) [9]. Equally, there are known interindividual and intraindividual variations in response to diet [8], and PN attempts to account for these, depending on the level of personalization (eg, sex, metabolic requirements, physiological difference, genome, and microbiome).

The internet has considerable potential to improve health-related food choices at low cost, via apps, for example. However, a review showed that none of the popular nutrition-related mobile apps reviewed had a decision engine capable of providing personalized dietary advice to the user [10]. Evidence from the Food4Me study indicated that web-based PN advice based on dietary intake (assessed using a validated Food Frequency Questionnaire (FFQ) with portion-size photographs [11]) was more effective in improving adherence to dietary advice and diet quality than standard population guidance [12]. Their decision tree for tailoring the advice was executed manually by the researchers and automated after the completion of the randomized controlled trial (RCT) [13]; however, this automated decision tree is not currently publicly available. The authors of this study are not aware of any similar web-based PN RCT delivered automatically [14].

To address this need, our research team developed a mobile web app capable of delivering automated PN advice (eNutri v1.0 [15-17]), which, to our knowledge, is the only app to deliver PN advice automatically. Dietary advice was delivered immediately after completion of a web-based FFQ. The advice was based on and derived according to adherence to an 11-item modified Alternative Healthy Eating Index (m-AHEI). This measure of diet quality was a UK-adapted version of the US 2010 Alternative Healthy Eating Index (AHEI), which was selected for its strong association with cardiovascular disease (CVD) and health [18-20].

### Objectives

The aim of this RCT (the EatWellUK study) was to evaluate the impact of the web-based PN advice tool, eNutri, on increasing diet quality in UK adults compared with generalized population dietary advice delivered on the web. This study tested the hypothesis that personalized dietary advice is more effective at eliciting beneficial dietary change than general dietary public health guidance.

## Methods

### Overview

The EatWellUK study was a randomized, controlled, parallel, single-blinded dietary intervention, which was delivered on the web and conducted by the Hugh Sinclair Unit of Human Nutrition (University of Reading, United Kingdom) between August 2017 and January 2018. It was designed to compare the impact of eNutri’s automated personalized food-based dietary advice with generalized population dietary advice (control) delivered on the web on change in diet quality (assessed by the m-AHEI score and the scores of its individual components; see Multimedia Appendix 1 [3,18,21-28] for details).

### Ethics Approval

The study was approved by the University of Reading (School of Chemistry, Food and Pharmacy) Research Ethics Committee (reference 13/17) and conformed with the Declaration of Helsinki. It was registered at ClinicalTrials.gov (NCT03250858).

### Recruitment and Consent

Participants were recruited from the Hugh Sinclair Unit of Human Nutrition’s volunteer database, University of Reading, mailing lists, social media (Facebook and Twitter), a university press release, web-based advertisements, and word of mouth. Interested parties received information with links to the consent form and participant information sheet hosted on the study website, where these documents were available on the home page for reading and downloading. The web-based account creation, using email and password, and the consent agreement were completed directly on the study website. It was made clear that participation was voluntary and that they were free to withdraw at any time without giving reason and without detriment. Participants were informed that they would need to complete web-based questionnaires at baseline, week 6, and week 12. There was no payment associated with participation, but to improve participant retention, all participants who completed the first set of questionnaires received an email regarding a prize draw (4 prizes of £50 [US $67.65] shopping vouchers were available) subject to the completion of the final questionnaire. All contact with participants was via the website or email.

### Screening and Randomization

Only participants aged ≥18 years were eligible to participate in the study. Screening was semiautomated in the eNutri web app, where a minimal set of exclusion criteria were applied automatically (not living in the United Kingdom, pregnant, lactating, receiving face-to-face nutrition services, lactose intolerance, food allergies, or diabetes). Other indications of potential exclusion were assessed by the researchers manually (self-report of health conditions, metabolic disorders, illness, medication, and specific dietary requirements), and in these cases, participants received an email to inform them of their eligibility.

As part of the screening form, participants were asked to report their age, sex, and highest level of education and how they heard about the study, selecting from the following options: email, Facebook, Instagram, Twitter, word of mouth, or other. Emails and social media links were created with customized URLs so that the app could also track the click source automatically [29,30]. Eligible participants were randomized automatically by the app using a random function [31] that generated a random number between 0 and 1. Depending on the value (lower or upper half of the interval), the participant was allocated to one of the two groups (PN or control). Allocation was concealed from the participants (single-blinded) who received advice at the same time points throughout the intervention.

### Study Protocol

The EatWellUK study protocol is summarized in Textbox 1. Following automatic randomization, participants were asked to complete the web-based FFQ [15] and Baecke PA questionnaire [32] and to provide their self-reported weight using the eNutri web app. These measures were repeated at weeks 6 and 12 of the intervention. General (control group; Multimedia Appendix 2) or personalized (PN group) advice was displayed immediately after completion of the FFQ at baseline (week 0) and week 6. All participants received the personalized recommendations at week 12 (upon completion of the study). The eNutri FFQ and advice are described more fully in the study by Zenun Franco et al [15] and Fallaize et al [16]; see also the *Intervention Groups* section.

**EatWellUK study procedure.**

**Procedure**

1. Web-based recruitment, providing the participant information sheet and consent form
2. Account creation via the study website
3. Web-based consent
4. Semiautomated screening (manual screening where analysis of text descriptions was required)
5. Participant’s characteristics (sex, age, height, and level of education)
6. Group allocation (randomization)
7. Weight, physical activity questionnaire, and Food Frequency Questionnaire
8. System Usability Scale questionnaire
9. Presentation of web-based advice
10. Personalized web-based advice evaluation (optional)
11. Follow-up questionnaire (optional)

Although participants were encouraged to complete the FFQ in 1 session, it was important to offer the possibility to save the FFQ in case of interruption or temporary internet disconnection. Hence, each food selection was saved individually (after the portion-size selection), and participants could return to the last saved food item when they logged in to the system again. Incomplete FFQs expired after 24 hours, after which the participant was required to start again to maintain the validity and accuracy of the FFQs.

The interval between FFQ completions was also managed by the app. The second FFQ was made available only after 41 days (1 day before the participant reached 6 weeks), and the third (and final) FFQ only after 83 days (12 weeks) to prevent completion of the FFQs too early. Reminders were sent by email a few days before the FFQs were due. If the participant logged in to the system during the intervals, a message was shown indicating the date when their next FFQ would be available.

Using eNutri, steps 1 to 9 (Textbox 1) were completed at baseline (week 0; ~20 minutes in total) [15]. This first completion of step 7 (weight, PA, and FFQ) served as baseline data. Steps 7 and 9 were presented again by eNutri in weeks 6 and 12. Step 8 (System Usability Scale questionnaire) was presented at baseline only; detailed methods and EatWellUK study data for the System Usability Scale questionnaire have been described in the study by Zenun Franco et al [15]. The optional step 10, requesting completion of a web-based report evaluation, was presented only at the end of the study. After an interval of almost 5 months after the study ended, a further follow-up questionnaire (step 11) was conducted using a web-based survey tool.

### Outcome Measures

#### Dietary Intake

Changes from baseline in dietary intake at end point were assessed via a graphical semiquantitative FFQ on the eNutri web app, which was based on a previously validated FFQ for a UK population [11]. The eNutri FFQ has been described previously [15]. The 2010 AHEI [18] was used as the foundation for (1) measuring the quality of the diet, (2) deriving the PN advice, and (3) quantifying changes in dietary intake. Some modifications were applied to the 2010 AHEI to adapt it to the UK dietary guidelines and to improve its use as the decision engine for the PN recommendations. The 11 food and drink components and scoring criteria for the m-AHEI can be found in Multimedia Appendix 1 [3,18,21-28]. The maximum component score was changed from 10 to 100, to facilitate data visualization and progress monitoring for the participant (details of the PN report are presented in the study by Fallaize et al [16]). Dietary intakes between the minimum (0 point) and maximum (100 points) criteria were scored using linear interpolation, with a positive slope for *healthy* components and a negative slope for *unhealthy* components, such that higher scores represented greater diet quality for every component. All the 11 individual components were weighted equally, and the overall score was presented to the participants as a percentage (ranging from 0 to 100) for ease of interpretation.

#### Weight, BMI, and PA Levels

Secondary outcome measures recorded via the eNutri web app included weight, BMI, and PA. Changes from baseline were measured for self-reported weight (kg) at end point. Change in weight was combined with height (collected in step 5) and reported as change in BMI (kg/m^2^). For PA levels, change was measured from baseline in self-reported PA (Baecke questionnaire) at end point.

As participants could be advised to either gain or lose weight to reach their ideal BMI range, an analysis of the change in BMI without considering the direction of the change (ie, increase or decrease) would not capture the effectiveness of the recommendation (ie, opposite variations across participants would cancel one another). Thus, the absolute difference from the current BMI to the ideal BMI was analyzed to determine if the personalized advice decreased this difference significantly, in comparison with the control group.

### Evaluation of Personalized Advice

Immediately after completion of the study at end point, the PN report was evaluated by the participants via 9 optional questions, also delivered via the eNutri web app, regarding the users’ perceived system effectiveness [33] and perceptions of its design. The first 6 questions were Likert questions, and the final 3 questions offered the possibility to write comments. As both groups received the PN report after completing the study, their responses were combined for this evaluation.

### Follow-up Questionnaire

To assess the long-term impact of the PN advice, a web-based follow-up questionnaire was administered via Online Surveys (Jisc Online Surveys) 4.6 months after the study ended, which invited all participants to provide feedback about eNutri and the advice they received. Those who responded and consented to participating were asked 32 questions, including Likert and multiple-choice questions. The primary purpose of these questions was to identify to what degree the PN advice had encouraged those in the PN group to improve their diets during the study and whether they were still following any aspects of their advice. Where they did not follow the advice, participants were also asked to identify their reasons for not doing so. In addition, the follow-up questionnaire included free-text boxes for the participants to write a short review of eNutri and comment on the advice they received. Although data for the control group were also obtained, these data are not presented here, as this group also received a PN report at the end of the study and, as such, their responses will likely be confounded.

### Intervention Groups

#### PN Intervention

The PN report received by this group via the eNutri web app [16] consisted of (1) the participant’s overall m-AHEI score (out of 100), (2) 3 diet messages, (3) feedback on BMI (including their ideal weight range), and (4) feedback on PA. The diet messages were tailored for each participant based on the 3 lowest m-AHEI component scores assessed with data from the FFQ, following a protocol published previously [15,16]. The components were presented as food-based recommendations; for example, if one of the lowest m-AHEI scores was *red or processed meat*, then the advice would use the FFQ data to highlight which individual foods in their diet were the highest contributors. Participants in the PN group were able to see a progress report after each subsequent FFQ (weeks 6 and 12). In the software version deployed in this study (eNutri v1.0), the inputs to the decision engine generating the PN feedback were a participant’s dietary data and sex.

The ideal weight range of the participants was based on their BMI calculation. A healthy BMI ranges from 18.5 to 25.0 kg/m^2^; hence, an ideal weight for a participant was presented as the midpoint at 21.75 kg/m^2^. Textual messages and visual representations in the app were also tailored according to BMI (eg, colored bars on the scale to represent the ideal weight range) [15,16].

The PA feedback was based on the Baecke questionnaire [32]. Participants were presented with their overall PA scores, followed by scores for the three categories (sports, leisure, and work), as defined by Baecke et al [32]. Scores were on a scale of 0 to 100, with higher scores representing greater levels of PA. Advice messages related to the sports and leisure categories were provided according to the participant’s score in each category. As it was deemed unlikely for participants to have much control over the nature of their activities at work, no personalized message regarding the work category was provided [15].

#### Control

The control group received generic healthy eating advice at baseline and week 6 via the eNutri web app (Multimedia Appendix 2). The report included 3 generalized healthy eating messages that were based on the m-AHEI components (baseline: *vegetables*, *free sugars*, and *polyunsaturated fatty acids*; week 6: *fruit*, *wholegrain products*, and *red or processed meat*). General advice was also provided on the importance of maintaining a healthy weight and attaining adequate PA (Multimedia Appendix 2); however, this was not tailored according to the participants’ BMI or reported PA levels. Following the final FFQ at week 12, the control group were provided with PN (intervention) advice. The UK Government’s Healthy Eating Recommendations were used as a basis for the component messages [34].

### Data Handling

As not every participant completed the eNutri questionnaires on the target dates of 6 and 12 weeks (some took longer to respond), only 2 questionnaires per participant were considered in the outcome analysis: baseline and the date closest to the target date of 12 weeks, referred to as end point. The effectiveness of the decision engine was captured in terms of users’ actual dietary change between baseline and end point, using the m-AHEI as the primary outcome measure.

Participants were excluded from the analysis if (1) their ratio of energy intake to basal metabolic rate (estimated using the equation of Henry [35]) exceeded standard cutoffs (men: <0.49, >2.79; women: <0.56, >3.21; n=14) [36]; (2) there was a large difference in energy intake between FFQs (>8000 kJ) without corresponding weight change (n=3), or (3) reported intakes of food groups were considered unfeasible in relation to maximum adult intakes reported in the National Diet and Nutrition Survey [37], such as 10 eggs or 1.2 kg of porridge daily (n=6).

### Statistical Analysis

Statistical analysis was conducted using Python StatsModel (version 0.11) [38]. Normality was tested using the D’Agostino-Pearson and Shapiro-Wilk tests [39], and, where necessary, m-AHEI component data were square root transformed. Treatment effects were determined based on the change from baseline between groups, where *P*≤.05 was considered significant. To account for mean lower-energy intake reported at the end of the study compared with baseline (PN: –1316, SD 2315 kJ/day; control: –726, SD 2549 kJ/day) in the absence of weight change, baseline energy intakes were included as a covariate for the m-AHEI analysis only. Secondary outcomes (PA and BMI) were adjusted for baseline values and presented as adjusted means [40]. Unless specified, data are presented as means (SDs).

Participants in the PN group received advice on specific m-AHEI components (based on their 3 lowest m-AHEI components). For analysis of change in individual m-AHEI components (eg, *fruit*), a smaller treatment effect is expected if the participant in the PN group did not receive advice for changing that specific component. Furthermore, the subgroup of participants in the control group with low scores for a specific component have greater room to improve their score (ie, greater distance to the maximum score) for that component than the group as a whole. To account for these factors, in addition to the treatment effects for the whole group, a treatment effect was also calculated for the subgroup of participants in the PN group who received personalized messages for a specific component, in comparison with participants in the control group who were matched in the sense that, based on their 3 lowest m-AHEI scores, they would also have received advice on that component had they been in the intervention group.

This RCT was powered based on the outcomes of the similar Food4Me study [12], comparing participants who received control advice with PN advice based on dietary intake only, expecting an increase of 6.5% (mean 49.58%, SD 9.51%; α=.05; power=0.8) in the m-AHEI (Food4Me consortium, unpublished data, October 2014). With these variables, the recruitment target was 274 participants, increasing to 330 participants when factoring a 20% dropout rate.

## Results

### Participants

A total of 438 participants created accounts in the eNutri web app. Table 1 presents which recruitment sources were reported by the participants and the results of the URL automatic tracking. Although sources were identified (self-reported) by 91.6% (401/438) of participants, the automatic URL tracking identified sources for just 61.6% (270/438) participants. The most frequently self-reported recruitment sources were email (164/438, 37.4%), Facebook (59/438, 13.5%), and Twitter (43/438, 9.8%).

**Table 1:** Recruitment sources as self-reported by all participants creating an account (N=438) and from automatic detection by the app.

Recruitment source	Self-report, n (%)	Automatic URL track, n (%)
Email	164 (37.4)	199 (45.4)
Facebook	59 (13.5)	26 (5.9)
Twitter	43 (9.8)	11 (2.5)
Instagram	0 (0)	0 (0)
Word of mouth	63 (14.4)	0 (0)
Other	72 (16.4)	34 (7.8)
Not available	37 (8.4)	168 (38.4)

**Figure 1:**
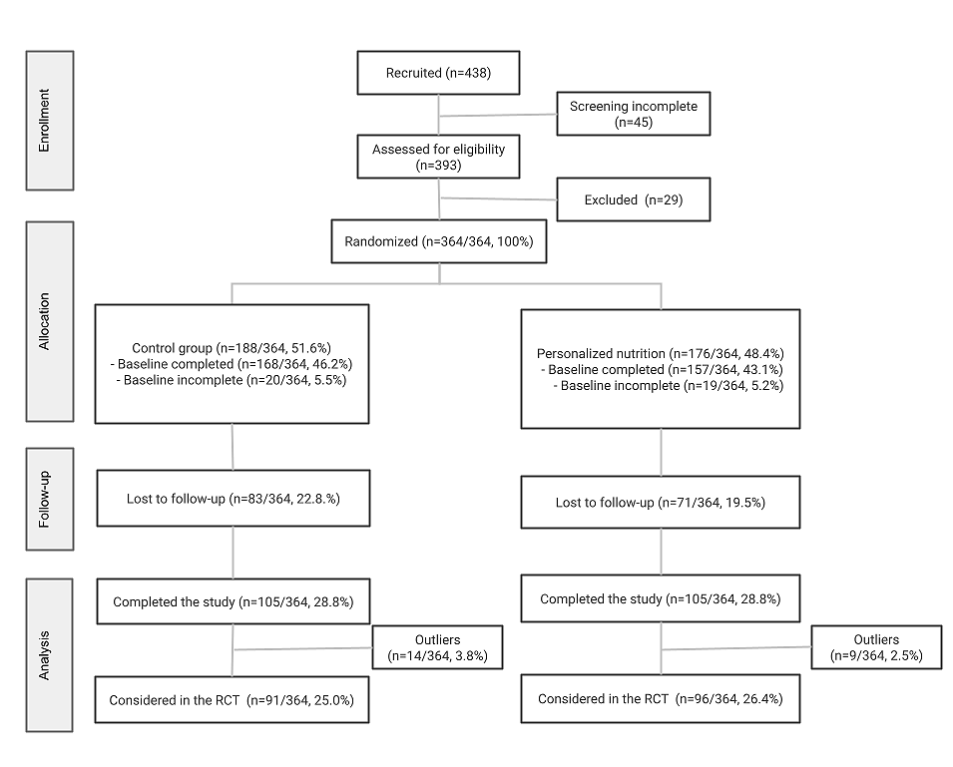
CONSORT (Consolidated Standards of Reporting Trials) flow diagram for the EatWellUK study. n values are expressed as percentages of the number of participants who were randomized (N=364). RCT: randomized controlled trial.

Of the 438 accounts, 393 (89.7%) completed the screening questionnaire. Of these 393, 29 (6.6%) participants were excluded owing to country of residence (n=6), medication use (n=8), or dietary restrictions, such as lactose intolerance (n=10) or food allergies (n=7). The remaining 83.1% (364/438) of the participants were automatically randomized by the app to either the control or PN group, although 10.7% (39/364) of these participants did not complete the baseline questionnaires (Figure 1).

Of the 325 participants who completed the baseline FFQ, 115 (35.4%) were lost to follow-up (ie, they did not complete the next FFQ), and 210 (64.6%) completed at least 1 additional FFQ, and these were considered in the RCT. Of these 210, 23 (11%) were removed as outliers for unfeasible dietary intakes. A total of 114 participants from the control (n=54) and PN (n=60) groups completed all 3 FFQs. At the end of the study, the participants were presented with an optional personalized web-based report evaluation questionnaire to provide feedback on the PN report. Of the 111 feedback forms received, 50 (45%) were from the control group and 61 (55%) from the PN group. These feedback responses were combined because all participants were given personalized reports at the end of the study, and no significant differences were found between the groups (data not shown). The baseline (week 0) characteristics of the participants included in the analysis after removal of outliers are presented in Table 2; no significant differences in sex, age, or educational attainment were observed between the intervention groups.

**Table 2:** Baseline characteristics of the EatWellUK study participants (N=187).

Characteristics		Total sample	Control group	Personalized nutrition group	*P* value	
Participants, n (%)		187 (100)	91 (48.7)	96 (51.3)		
**Sex, n (%)**						.07
	Female	157 (84)	81 (43.3)	76 (40.6)		
	Male	30 (16)	10 (5.3)	20 (10.6)		
Age (years), mean (SD; range)		43.2 (15.0; 18-85)	42.8 (14.0; 20-82)	43.5 (15.9; 18-85)	.76	
**Highest level of education, n (%)**						.19
	Less than secondary	0 (0)	0 (0)	0 (0)		
	Secondary	20 (10.6)	13 (6.9)	7 (3.7)		
	College	21 (11.2)	11 (5.8)	10 (5.3)		
	Undergraduate	64 (34.2)	25 (13.4)	39 (20.9)		
	Postgraduate	82 (43.9)	42 (22.5)	40 (21.4)		

### Primary Outcomes Evaluation

Considering the protocol for selecting the end point FFQ (ie, the one closest to week 12), the trial resulted in an average interval between FFQs of 10.8 weeks. The analysis by group confirmed that the intervals were equivalent across the control (10.7 weeks) and PN (10.8 weeks) groups. The results for the overall changes in m-AHEI scores from baseline to end point (10.8 weeks) are presented in Table 3.

**Table 3:** Effects of the EatWellUK intervention on the m-AHEI^a^ component scores, considering all the participants in the control (n=91) and PN^b^ (n=96) groups.^c^

m-AHEI variables		Baseline, mean (SD)		Adjusted Δ,^d^ mean (SD)		Treatment effect, Δ PN–Δcontrol (95% CI)^d^	*P* value
		Control	PN	Control	PN		
Overall m-AHEI score		58.9 (12.3)	56.3 (11.5)	–0.4 (2.3)	3.1 (2.1)	3.5 (1.19 to 5.78)	.003
**Positive components**							
	Vegetable score	68.0 (26.0)	61.2 (28.3)	–4.0 (8.4)	–7.1 (9.4)	–3.2 (–9.31 to 3.01)	.32
	Fruit score	67.0 (31.0)	60.6 (34.8)	–5.7 (6.8)	–3.7 (7.5)	2.0 (–4.32 to 8.22)	.54
	Whole grain score^e^	43.5 (35.4)	35.5 (33.9)	–0.6 (1.2)	–0.3 (1.1)	0.3 (–0.45 to 0.95)	.48
	Dairy product score	87.7 (27.8)	95.0 (18.1)	–0.9 (13.6)	0.4 (8.9)	1.3 (–4.96 to 7.52)	.69
	Nuts and legume score^e^	47.3 (38.3)	26.7 (33.1)	0.3 (1.4)	1.2 (1.2)	0.9 (0.03 to 1.76)	.04
	Healthy fats score	53.4 (17.7)	50.8 (16.7)	0.7 (7.5)	–1.2 (7.1)	–1.9 (–6.36 to 2.55)	.40
	Oily fish score	63.0 (41.7)	69.2 (38.0)	–2.1 (17.3)	3.7 (16.0)	5.8 (–3.72 to 15.3)	.23
**Negative components**							
	Free sugars score	44.3 (27.7)	50.5 (26.8)	–2.3 (8.9)	3.9 (8.5)	6.1 (–0.33 to 12.6)	.06
	Red and processed meat score^e^	27.5 (36.6)	24.4 (36.1)	0.4 (0.9)	1.2 (0.9)	0.8 (0.05 to 1.58)	.04
	Salt score	55.9 (34.4)	57.0 (30.7)	7.8 (17.1)	14.1 (15.3)	6.3 (–0.90 to 13.5)	.09
	Alcohol score	90.2 (27.2)	88.7 (27.8)	2.8 (15.6)	3.5 (15.4)	0.6 (–5.15 to 6.41)	.83

^a^m-AHEI: modified Alternative Healthy Eating Index.

^b^PN: personalized nutrition.

^c^Scores are reported on a scale between 0 and 100, where higher scores reflect greater diet quality.

^d^Change from baseline at end point. Data are presented as adjusted means with the baseline energy intakes as a covariate [40].

^e^Square root transformation.

Compared with that for the control group, the treatment effect observed in the overall m-AHEI score for the PN group was 3.5 out of 100 (95% CI 1.19-5.78), which was reached statistical significance (*P*=.003). A statistically significant improvement in *nuts and legumes*, and *red and processed meat* scores were also observed in the PN group compared with the control group during the intervention period (*P*=.04), reflecting an increased intake of nuts and legumes and reduced intake of red and processed meat.

All the participants in the PN group (n=96) received feedback regarding their overall m-AHEI score and were able to see the progress report with all the individual m-AHEI scores; however, the focus of the advice was on just 3 components [15].

The outcomes presented in Table 3 do not consider whether a specific participant received a personalized message for that component but rather how the individual m-AHEI components were affected by the intervention as a whole. Table 4 shows the component messages presented to the PN group during the intervention (ie, the 3 m-AHEI components with the lowest scores), and the *matched* participants from the control group who would have received those messages had they been in the PN group. The distribution presented in the final column of Table 4 (total messages) gives an indication of the components for which the EatWellUK cohort were most in need of improvement. *Red and processed meat*, *nuts and legumes*, and *whole grains* were the components most frequently presented by the eNutri app algorithm across both groups for having the lowest m-AHEI scores, whereas *dairy products*, *alcohol*, and *vegetables* were presented in <10% of cases for both groups, suggesting these components required least improvement in the participant’s diets.

**Table 4:** Frequency of healthy eating messages presented to the PN^a^ group (n=96) and matched messages in the control group (n=91) at baseline when only messages for the three components with the lowest scores were considered.^b^

	Matched control messages, n (%)	PN messages, n (%)	Total messages, n (%)
Red and processed meat	60 (32.1)	66 (35.3)	126 (22.5)
Nuts and legumes	42 (22.5)	70 (37.4)	112 (19.9)
Whole grains	39 (20.9)	48 (25.7)	87 (15.5)
Salt	27 (14.4)	23 (12.3)	50 (8.9)
Free sugars	30 (16)	17 (9.1)	47 (8.4)
Oily fish	26 (13.9)	18 (9.6)	44 (7.8)
Fruits	14 (7.5)	15 (8)	29 (5.2)
Healthy fats	13 (7)	10 (5.3)	23 (4.1)
Vegetables	7 (3.7)	9 (4.8)	16 (2.9)
Alcohol	7 (3.7)	8 (4.3)	15 (2.7)
Dairy products	8 (4.3)	4 (2.1)	12 (2.1)
Total messages	273 (48.7)	288 (51.3)	561 (100)

^a^PN: personalized nutrition.

^b^Components are ordered by the total number of healthy eating messages that were (personalized nutrition group) or would have been (control group) presented to participants. The personalized nutrition and control group data are presented as a contribution to the total sample of messages produced by eNutri. Because each participant (n=187) received or would have received 3 messages from eNutri, the total number of messages is 561.

The treatment effect on participants in the PN group who received personalized messages for a specific component was also calculated in comparison with the matched participants in the control group, as shown in Table 5. Although participants in the PN group displayed greater score improvements across all m-AHEI components, except *vegetables*, significant treatment effects were only observed for *salt* (+18.3; *P*=.04) and *alcohol* (+51.4; *P*=.03) in the PN group compared with that in the control group, reflecting a significantly greater reduction in intake of these components following PN intervention.

**Table 5:** Changes in the m-AHEI^a^ component scores from baseline to end point for participants in the PN^b^ group (n=96) who received these specific component messages and the matched participants in the control group (n=91).

m-AHEI component		Matched control				PN group			Treatment effect, Δ PN–Δcontrol (95% CI)^c^			*P* value
		Value, n (%)^d^	Baseline, mean (SD)	**Δ** (SD)^c^	Value, n (%)^d^		Baseline, mean (SD)	**Δ** (SD)^c^				
**Positively scored components**												
	Nuts and legumes^e^	42 (46.2)	19.1 (17.7)	1.5 (0.8)	70 (72.9)		13.9 (19.2)	2.3 (0.8)		0.8 (–0.41 to 1.98)	.20	
	Whole grains^e^	39 (42.9)	16.8 (16.3)	0.2 (0.3)	48 (50)		11.1 (13.9)	1.0 (0.3)		0.8 (–0.27 to 1.78)	.15	
	Oily fish	26 (28.6)	7.4 (14.6)	23.8 (7.7)	18 (18.8)		10.3 (15.6)	31.7 (8.3)		7.9 (–15.2 to 31.0)	.49	
	Fruits	14 (15.4)	26.1 (18.8)	1.1 (2.5)	15 (15.6)		18.3 (12.2)	5.0 (3.1)		3.9 (–10.0 to 17.9)	.57	
	Healthy fats	13 (14.3)	46.8 (15.7)	8.0 (8.1)	10 (10.4)		38.1 (14.9)	8.2 (8.7)		0.2 (–13.3 to 13.6)	.98	
	Vegetables	7 (7.7)	40.0 (7.5)	10.5 (4.0)	9 (9.4)		26.0 (15.2)	3.9 (7.1)		–6.7 (–36.1 to 22.8)	.63	
	Dairy products	8 (8.8)	20.1 (13.8)	8.2 (21.0)	4 (4.2)		22.8 (14.1)	25.7 (17.5)		17.5 (–47.0 to 82.0)	.55	
**Negatively scored components**												
	Red and processed meat^e^	60 (65.9)	4.3 (8.9)	0.9 (0.6)	66 (68.8)		3.3 (8.3)	1.9 (0.6)		1.0 (–0.06 to 2.00)	.06	
	Salt	27 (29.7)	14.1 (18.3)	31.5 (16.6)	23 (24)		25.7 (24.5)	49.8 (22.8)		18.3 (1.18 to 35.5)	.04	
	Free sugars	30 (33)	21.6 (24.8)	7.0 (9.2)	17 (17.7)		9.9 (14.5)	19.3 (5.4)		12.3 (–3.40 to 28.0)	.12	
	Alcohol	7 (7.7)	8.4 (17.6)	–1.5 (36.8)	8 (8.3)		11.4 (20.7)	49.9 (38.4)		51.4 (4.93 to 97.8)	.03	

^a^m-AHEI: modified Alternative Healthy Eating Index.

^b^PN: personalized nutrition.

^c^Change from baseline at end point. Data are presented as adjusted means with baseline energy intakes as a covariate.

^d^Values represent percentage of intervention group (control and intervention) who received component messages.

^e^Square root transformation.

### Secondary Outcomes Evaluation

As both the PN and control participants received advice on weight and PA (albeit in different formats), analysis of matched participants was not required. Absolute BMI was not affected by the treatment (Table 6). The mean distances to the ideal BMI decreased slightly (ie, BMI improved) in the PN group (–0.09 kg/m^2^) with no change in the control group, but this improvement was not statistically significant from the control group (*P*=.37). A number of participants in the control (n=13) and PN (n=21) groups reported the same weight at the end point and baseline. Although a significant improvement in Work score was reported in the PN group (*P*=.02) compared with the control group, there were no significant differences in change in overall Baecke score between the groups (*P*=.70).

**Table 6:** Changes in BMI and PA^a^ level (Baecke) score from baseline to end point for participants in the control (n=91) and PN^b^ (n=96) groups.^c^

			Baseline, mean (SD)				Adjusted, Δ (SD)					Treatment effect, Δ PN–Δcontrol (95% CI)			*P* value			
			Control (n=91)		PN (n=96)		**Δ**Control (n=91)		**Δ**PN (n=96)									
**BMI (kg/m^2^)^d^**																		
	Absolute BMI	24.2 (4.4)		24.8 (4.4)		–0.1 (0.1)		–0.1 (0.1)		0.0 (–0.23 to 0.18)			.79					
	Ideal BMI distance	3.5 (3.6)		3.7 (3.9)		0.0 (0.2)		–0.1 (0.2)		–0.1 (–0.29 to 0.11)			.37					
**PA (Baecke) score^e^**																		
	Overall score	53.7 (9.6)		51.3 (9.5)		0.3 (1.7)		0.6 (1.6)		0.3 (–1.26 to 1.87)			.70					
	Leisure score	60.1 (13.1)		59.5 (13.3)		–2.1 (3.3)		–1.0 (3.5)		1.2 (–1.26 to 3.62)			.34					
	Sports score	55.8 (20.6)		49.5 (18.9)		2.9 (4.3)		0.4 (3.9)		–2.5 (–5.83 to 0.83)			.14					
	Work score	45.3 (11.6)		45.2 (10.4)		0.4 (1.6)		2.3 (1.3)		2.0 (0.29 to 3.63)			.02					

^a^PA: physical activity.

^b^PN: personalized nutrition.

^c^Values presented as adjusted means.

^d^Presented as absolute variation and distance to the ideal BMI (21.75 kg/m^2^).

^e^Values are reported on a scale between 0 and 100.

### Personalized Web-Based Report Evaluation

Both the control and PN groups were presented with the PN report following the final FFQ, after which 108 provided complete feedback on the PN report. Most participants (95/108, 88%) did not report difficulty in understanding the report (question 1; Table 7). Of those who did, comments largely related to the stages before the PN advice itself (n=8), including minor issues related to the FFQ (n=3), Baecke questionnaire (n=3), and difficulties finding the link to the web-based report (n=2). Comments relating to the PN advice covered the desire to see the scientific evidence for the recommendations (ie, details of the m-AHEI score calculations) (n=1) and disagreement with the personalized advice received (n=4).

**Table 7:** Qualitative user feedback for the open questions related to the personalized report (N=108).

Question	Yes, n (%)	No, n (%)
Question 1: Was there anything in the report that you found particularly difficult to understand?	13 (12)	95 (88)
Question 2: Do you need additional information to help you make changes to your diet at this moment?	11 (10.2)	97 (89.8)
Question 3: Do you have any further comments regarding the feedback you received?	16 (14.8)	92 (85.2)

In response to question 2 (Table 7), 83% (5/6) of the comments provided were about barriers to healthy eating (eg, “more time to prepare meals”), and 1 participant requested more explanation of the advice (“If you want me to follow advice, I would like to understand the basis”). Of the 14 comments received in response to the third question, 3 (21%) related to the FFQ, 5 (36%) were about the limitations of the PA feedback (eg, “I do not think the report is a reflection on my sporting activity”), and the other 6 (43%) were about the diet recommendations, most of which (4/6, 67%) mentioned their partial disagreement with some of the diet advice (eg, “I do not agree with the advice to increase dairy foods. This is a very narrow view of the full picture,” and “I have too much salt and meat, but I don’t think I do”).

**Figure 2:**
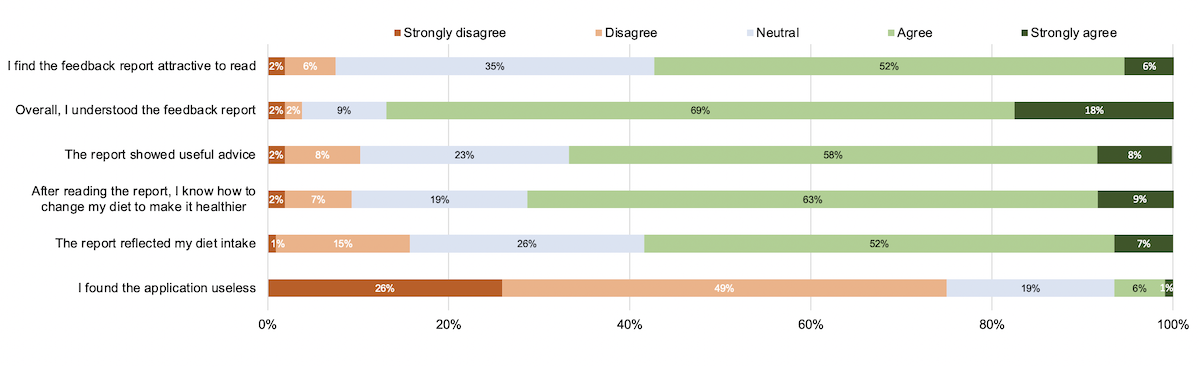
User evaluation of the web-based personalized nutrition report using a Likert scale (N=108). Inconsistencies in the sum of percentages is due to the rounding of the percentages.

The results of the questions related to the quality of the report design and the perceived effectiveness of the recommendations [33] are shown in Figure 2 using a Likert scale. Although most participants (94/108, 87%) agreed to understanding the feedback, less reported (*agree* and *strongly agree*) knowing how to change their diet following feedback (77/108, 71.2%) and finding the advice useful (72/108, 66.7%). In addition, 15.7% (17/108) of participants felt that the report did not reflect their dietary intake, and 7% (7/108) reported finding the eNutri app useless.

### Follow-up Questionnaire

In total, 82 participants returned to complete the follow-up questionnaire that was administered 4.6 months after the main study ended; 42 (51%) of these were in the PN group (n=96) and included in the analysis. The mean follow-up time was 5.9 months (range 4.6-7.6 months, SD 0.65).

**Figure 3:**
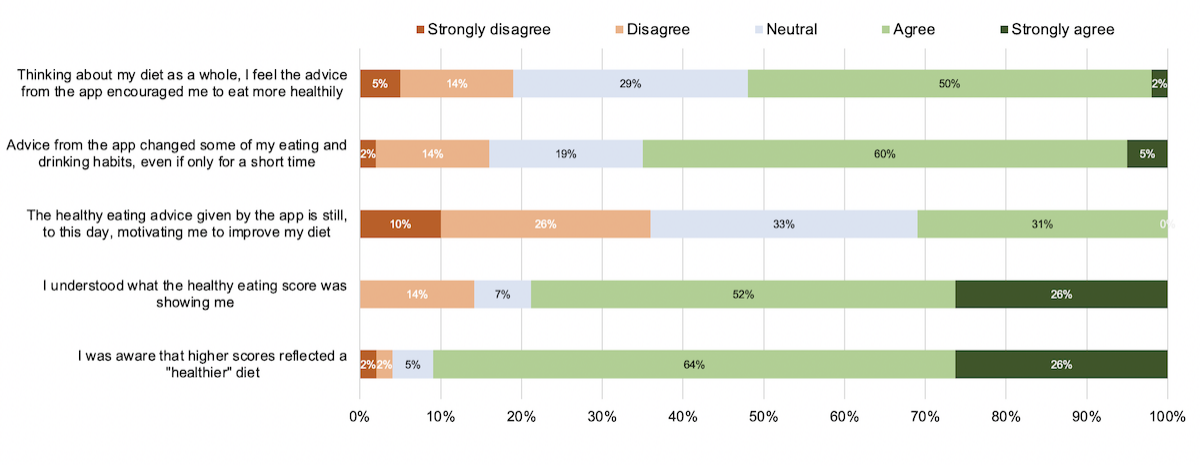
Follow-up questionnaire responses in the personalized nutrition group using a Likert scale (N=42). Inconsistencies in the sum of percentages is due to the rounding of the percentages.

Over half of the PN participants agreed or strongly agreed that the advice encouraged them to eat more healthily (22/42, 52%) and that it changed some of their eating or drinking habits (27/42, 65%), with almost one-third (13/42, 31%) claiming that the advice was still motivating them to improve their diet 5.9 months (mean follow-up) after the study had ended (Figure 3). However, when asked, “Compared to before you started the study, are you still following ANY of the healthy eating advice in your diet now, no matter how small the changes?” this value was much greater, with 64% (27/42) responding “Yes” compared with 7% (3/42) “No” and 29% (12/42) responding “I did not make any changes to my diet whilst using the app.” It should be noted that during the follow-up questionnaire, 74% (31/42) of the participants indicated they were “already motivated” to make changes to their diet before starting the study. Furthermore, the inclusion of their m-AHEI (*healthy eating*) score in the personalized advice was generally understood (33/42, 79% agreed or strongly agreed).

The three highest rated reasons why participants did not follow the advice given were “The recommended foods didn’t fit into my usual meal plans/recipes” (20/42, 47% agreed or strongly agreed), “The advice was too general and unrelated to my diet” (16/42, 38%), and “The advice was not suited to my lifestyle” (13/42, 31%). The reasons most participants disagreed or strongly disagreed with were “I was concerned my weight would change” (31/42, 74%), “I wasn’t willing to try new foods” (31/42, 74%), and “I won’t change certain aspects of my diet, regardless of the advice” (30/42, 71%). When asked to rate which features would be helpful additions to eNutri, the top three were “More information about the most positive aspects of my diet” (25/42, 60% rated this *extremely* or *very* helpful), “Advice on the vitamins and minerals to improve in my diet” (27/42, 64%), and “Advice on my overall energy (calorie) intake” (23/42, 55%).

Participants were also asked to write a review of the app, in which some positive comments were provided, such as “The dietary advice was relevant and easy to implement into my diet, and was evidence based so felt trustworthy. From just a short questionnaire, it was able to give personalised recommendations and practical ways to implement them into my diet. A great resource for anyone wanting to adopt a healthier dietary pattern” and “A few of the results I got from the app were surprising and encouraged me to eat differently—particularly eating more whole grains and eating less processed meat—neither of which were diet modifications that had ever occurred to me.”

## Discussion

### Principal Findings

This RCT was designed primarily to test whether eNutri’s personalized food-based dietary web-based advice, using the m-AHEI as the foundation of the decision engine, was more effective than generalized population advice for motivating beneficial dietary change. The significant treatment effect (3.5 points in the m-AHEI scale) represented an increase of 6.1% in the mean baseline m-AHEI score of 57.5 points. This result supports the hypothesis that the eNutri app is an effective web-based tool for PN advice for UK adults.

A total of 2 m-AHEI component scores, *nuts and legumes* and *red and processed meat*, significantly increased, indicating beneficial dietary change. All component scores increased more in the PN group than in the control group, except for *vegetables*, indicating that the personalization could potentially have reached significance with more participants. The reduced population consumption of red and processed meats has become a priority for many health organizations (eg, the World Health Organization and World Cancer Research Fund) owing to the observed reduction in colorectal cancer risk following reduced intake (particularly in high consumers) and the significant impact of these items on food-related greenhouse gas emissions impact [41].

The most frequently presented components, representing dietary intakes that deviated most from the dietary recommendations in the m-AHEI, were *red and processed meat*, *nuts and legumes*, *whole grains*, *salt*, and *sugars*. Targeted analysis of m-AHEI components, based upon those presented to participants or matched controls, revealed significant decreases in *salt* and *alcohol* intake (ie, greater m-AHEI scores) following PN advice as compared with control advice. However, *alcohol* was presented in only 2.7% (8/187) of cases; thus, further data are required to confirm these findings with larger sample sizes.

The observed change in diet quality between the 2 groups was 3.5 points out of 100 (or 3.9 points out of 110 before scaling down). Although relatively small, long-term dietary changes of this magnitude are likely to have a positive impact on health if continued over time. For example, the UK Whitehall II study demonstrated that an increase of 1 SD (equivalent to 9.8 points out of 110) in the 2010 AHEI (on which the m-AHEI was based) was associated with a reduced risk of all-cause (–22%) and CVD mortality (–20%) after a 22-year follow-up [42]. Therefore, an increase in diet quality by 3.9 points maintained long term could potentially reduce the risk of CVD mortality to some degree, although further investigation of the m-AHEI score is warranted. Similarly, findings from the UK Caerphilly Prospective study reported that the risk of stroke was 17% lower for every 1 SD increase in the 2010 AHEI (equivalent to 11 points) in middle-aged men after a mean follow-up of 16.6 years [43]. There is an absence of data examining the impact of changes in diet quality on risk factors of disease in the shorter term, and this is an area worthy of further work.

Before the EatWellUK study, the most closely related and important work was the Food4Me study [44], in which 1269 participants from 7 European countries completed a 6-month PN study. The Food4Me study used the Healthy Eating Index (HEI) [45], which was the basis for the AHEI [18], as a secondary outcome measure of diet quality. Their treatment effect on the overall HEI was 1.27 points out of 100 (95% CI 0.30-2.25; *P*=.01) at 6 months, suggesting a significant improvement in diet quality following PN advice. Participants randomized to receive PN advice were reported to consume less red meat, salt, saturated fat, and energy and also increased their folate intake [12]. Although statistically significant, the increase in HEI in Food4Me was relatively small, confirming the challenge to encourage healthier diets and the need of similar studies.

A systematic review of remotely delivered dietary interventions (n=26) using self-monitoring or tailored feedback also concluded a significant, but small (and at risk of bias), positive effect on dietary change [14]. A total of 51 dietary outcomes were analyzed in the 23 interventions considered in the meta-analysis, resulting in an average of 2.2 dietary outcomes per intervention. The most popular ones were *fruits*, *vegetables*, and *fat*, and only 3 interventions targeted >4 dietary outcomes. This review also considered interventions delivered over the phone or offline media (eg, printed reports and CD-ROMs). Only 7 interventions used modern web-based methods, such as websites or apps. The differences in the dietary outcomes make the comparisons more difficult, especially because the changes in some dietary outcomes may affect other components not measured during the intervention; for example, a decrease in alcohol consumption may be associated with an increased consumption of sugar-sweetened beverages, owing to the dynamic aspect of diets.

A more recent systematic review (n=6) on the use of mobile apps to improve nutrition behaviors reported significant improvements in some objectives (eg, weight status) for 67% (4/6) the trials included [46]. Similar to eNutri, most apps (83%) in the review used self-assessment for feedback and monitoring. Social support in the form of personalized coaching calls [47] and a phone call and social networking feature [48] were used in 33% (2/6) of the studies. However, as noted by the review’s authors, this requires significant financial input [49]. Regarding deployment costs, this version of eNutri can be deployed in Google Firebase (database and hosting) using the free plan, allowing reproducibility and scalability.

It should be noted that this study was powered to measure the overall m-AHEI treatment effect in all the participants but not the individual components. The fact that individual m-AHEI scores started from different baselines and were presented to the participants more or less frequently according to the participant’s dietary intake makes it more difficult to reach statistical significance. For example, some m-AHEI components, such as *dairy products* and *alcoho*l started with mean baseline values close to the best possible score (≥88 out of 100) and were thus presented to small numbers of participants. To test the significance of the personalization of these diet messages, a much larger RCT would be necessary, which is viable over the internet.

At follow-up (mean 5.9 months after intervention), 64% (27/42) of PN participants agreed that the advice from the app had prompted them to change their eating or drinking habits and that they were still following some (*any*) of the advice. It is important to note that this sample represented only 44% (42/96) of the PN completers; therefore, these data should be interpreted with caution. However, maintenance of lifestyle changes, including dietary change, following intervention is arguably one of the greatest challenges that researchers and clinicians face; thus, it is encouraging to see reported benefits at follow-up in this study. A key consideration for personalized or precision nutrition, particularly using apps or wearables, is long-term user engagement and maintenance [50]. In a cross-sectional survey (n=217) designed to identify the impact of diet- and nutrition-related apps of health behavior change, both app engagement (*P*<.001) and theory-related constructs (*P*<.001) were positively associated with diet-related behavior change [51], with the authors recommending integration of appropriate theoretical constructs for health behavior change into app development [51]. Extensive user feedback, such as that collected in this study, is an important step to understanding how to drive future development.

The evaluation of the PN report showed that participants largely understood the report and were confident about the changes they were advised to make in their diets. Although users and nutrition professionals were consulted in the design and composition of the PN feedback, a small number of participants reported disagreeing with the advice provided (n=4) [16]. However, there is scope to enable user interaction within the app allowing the user to manipulate the advice to focus on the aspects they feel more capable of addressing, such as the choice to select which of the *top* components recommended for them they are prepared to change (ie, goal setting). There was also good acceptance of the content and design of the report, although the data suggested that the visual appearance (attractiveness) of the report could be improved, suggesting that further improvements in its design may be necessary. Of importance, most of the participants reported that they found the advice helpful.

In our study, a minimal decrease in the distance to the ideal BMI of 0.09 kg/m^2^ was seen in the PN group at 12 weeks; however, this did not significantly differ from the change observed in the control group. A minimal decrease was expected, as the intervention was primarily targeted to healthy eating and relatively short in duration. In Food4Me, the researchers also reported no significant effect of personalized advice on BMI (–0.24 kg/m^2^) relative to a control group [12], but it is difficult to compare the effectiveness on BMI because the authors did not report the distance to the ideal BMI, as proposed by this research. There was also no benefit of PA feedback based on the Baecke questionnaire, compared with the control advice in this study. It may confirm that more robust and personalized PA trackers, such as accelerometers, GPS, or pedometers, may be necessary for delivering effective interventions to increase PA.

### Limitations

The power calculation for this study was based on the expected increase in the overall m-AHEI score. Because of recruitment challenges and high dropout rate (35.4%), the planned number of participants (n=274) was not reached, with 187 participants providing at least two valid FFQs. However, it is worthy of note that statistically significant differences in our primary outcome (m-AHEI score) were identified between the intervention and control groups. Additional studies with more participants, considering the baseline values (eg, by randomizing according to baseline diet quality) and distribution of messages may be necessary if the individual m-AHEI components are to be analyzed. Therefore, where advice on a particular component was delivered to only relatively few participants, such as *dairy products*, the effect of the advice on the component should be read cautiously considering the large CIs described. Although high, our dropout rate is within the range reported in a systematic review of engagement with remote measurement technology for managing health outcomes (0%-44%) [52]. Higher dropout rates appear synonymous with remote interventions, but there is scope to mitigate these by exploring user experiences (eg, barriers to use). The review notes the following as key drivers of engagement and re-engagement: health status, usability, convenience, accessibility, perceived utility, and motivation [52]. In the short term, more realistic dropout estimates should be included in sample size calculations for future studies.

This study used a modified US-based diet quality score for derivation of PN recommendations. However, the development of a diet quality score that aligns with the UK Eatwell Guide is warranted for future delivery and evaluations of dietary interventions.

Although the design of the diet messages followed the same protocol for each component [16], some messages were presented to only a few participants (eg, for *dairy products*); thus, the reported understanding of the report should not be generalized to all the textual diet messages. The fact that diet and weight were self-reported on the web may have impacted on the results, although dietary assessment was based on a previously validated FFQ [11]. Some participants may not have had weighing scales at home or were not able to weigh themselves for the subsequent app visits, and participants may have re-entered the original value without taking a new measurement. This may explain why 24 participants reported no change in weight during the study, which may have impacted the lack of statistical significance for changes in BMI. A face-to-face validation study (n=140) performed after the Food4Me study found that BMI was significantly lower (–0.29 kg/m^2^; *P*<.001) for self-reported data, although BMI was still classified correctly in 93% of cases [53]. Underreporting bias for weight and BMI has also been reported previously [54,55].

A further limitation is that the FFQ was not repeated at the follow-up, which would have allowed the evaluation of longer-term dietary changes more accurately. This was to reduce the participant burden and address the low response rates in the main study. Instead, adherence to the dietary advice was self-reported in a questionnaire that may be subject to reporting bias. Moreover, because both groups received PN dietary advice at the end of the study, it was not possible to quantitatively compare the 2 groups at the follow-up.

### Conclusions

This novel study presented the treatment effects of a 12-week web-based RCT with 187 participants, which, to our knowledge, is the second largest web-based dietary intervention in the UK and the only one to deliver PN advice automatically [14]. This study aims to measure the effectiveness of a novel web-based PN advice tool (eNutri), using a modified version of the AHEI (a measure of diet quality) as the foundation of the decision engine to deliver web-based personalized food-based dietary advice. The results show that the design and protocol followed by the PN group in this study motivated greater change to follow a healthier diet relative to generalized dietary advice, as evaluated by an increase in diet quality. It is anticipated that the use of the eNutri app could contribute to improved diet quality if used more widely within the United Kingdom. The user evaluation of this study, via the web-based report evaluation and follow-up questionnaires, is important to improve eNutri and similar apps to motivate people to use them and follow the personalized advice given. The design principles and algorithms of eNutri can be used and improved by other researchers and institutions interested in web-based PN advice; the eNutri 1.0 web app was made publicly available under a permissive open-source license [56]. Larger studies evaluating the longer-term impact of automated PN interventions which include objective assessment of dietary intake and health outcomes, are recommended. This work represents an important landmark in the field of automatically delivered web-based dietary interventions, particularly those that are personalized to individual users.

## Appendix

Multimedia Appendix 1 Modified Alternative Healthy Eating Index components and scoring criteria.

Multimedia Appendix 2 Nutrition, weight, and physical activity advice received by the control group participants.

Multimedia Appendix 3 CONSORT-eHEALTH checklist (V 1.6.1).

## Abbreviations

AHEI: Alternative Healthy Eating Index
CVD: cardiovascular disease
FFQ: Food Frequency Questionnaire
HEI: Healthy Eating Index
m-AHEI: modified Alternative Healthy Eating Index
PA: physical activity
PN: personalized nutrition
RCT: randomized controlled trial
