# Supplementary material for: Effectiveness of Web-Based Personalized Nutrition Advice for Adults Using the eNutri Web App: Evidence From the EatWellUK Randomized Controlled Trial
Source: J Med Internet Res. 2022 Apr 25;24(4):e29088. doi: 10.2196/29088 (PMC9154737; doi:10.2196/29088)
Supplement: Multimedia Appendix 1 [file jmir_v24i4e29088_app1.docx]

**Supplementary Table 1. Modified Alternative Healthy Eating Index (m-AHEI) components and scoring criteria^a^**

| **Component name (name presented to the app user)** | **Portion/drink size** | **Scoring criteria units** | **Criteria for**  **minimum score (0)** | **Criteria for**  **maximum score (100)** |
| --- | --- | --- | --- | --- |
| Vegetables^b^ | 80 g vegetables | portions/d | 0 | ≥5 |
| Fruits^b^ | 80 g fruit and/or 30 g dried fruit | portions/d | 0 | ≥4 |
| Whole grains | ~15-20 g whole grains per dry weight serving^c^ | g/d | 0 | ≥75 (women) or ≥90 (men) |
| Dairy products^d^ | 200 ml milk, 35 g cheese and/or 125 g yogurt | portions/d | 0 | ≥3 |
| Nuts and legumes^e^ | 28 g nuts and/or 57g legumes | portions/d | 0 | ≥1 |
| Polyunsaturated fats (Healthy fats) | n/a | % of total energy | ≤2 | ≥10 |
| Long-chain n-3 polyunsaturated fats (Oily fish)^f^ | n/a | mg/d | 0 | ≥250 |
| Free sugars (Sugar)^g^ | n/a | % of total energy | ≥15 | 0 |
| Red and processed meat^h^ | 113 g red meat and/or 28 g processed meat | portions/d | ≥1.5 | ≤0.03 (<1 serving/month) |
| Sodium (Salt)^i^ | n/a | mg/d | 4195 | 1425 |
| Alcohol^j^ | 113 g wine, 340 g beer, lager or cider, and/or 43 g spirits | drinks/d | ≥2.5 (women) or ≥3.5 (men) | ≤1.5 (women) or ≤2.0 (men) |

^a^ Adapted from AHEI-2010 for a UK population ^[18]^.

^b^ Includes contribution from composite dishes, but not pure fruit/vegetable juice or smoothies.

^c^ Typical wholegrain content per serving (e.g., oats, wholemeal bread, wholemeal pasta or brown rice).

^d^ Not part of AHEI-2010 (replaced “trans fatty acids” component). The original “Trans Fat” component was excluded due to the significant reduction in trans fatty acids in food products in the UK, simulations with data from a prior study indicated that the vast majority of participants would meet the UK recommendation of <2% of total energy from trans fatty acids and as such would score very highly. The “dairy products” component was introduced in line with the UK’s Eat Well Guide (“try to have some milk and dairy food (or dairy alternatives) – such as cheese, yoghurt and fromage frais” ^[3]^ and Western European guidelines, such as the Netherlands (“take a few portions of dairy produce daily, including milk or yogurt”) ^[21]^ and France (“consume foods that are rich in calcium (mainly dairy products)”) ^[22]^.

^e^ Equivalent to 1 oz (28g) nuts and 2oz (57g) legumes.

^f^ Sum of eicosapentaenoic acid and docosahexaenoic acid intakes.

^g^ AHEI-2010 “sugar-sweetened beverages and fruit juice (serving/d)” component was modified to “Free sugars (% of total energy)” to capture all dietary sources of free sugar and meet the recent recommendations in the UK ^[23][24]^.

^h^ Equivalent to 4 oz (113g) red meat and 1oz (28g) processed meat. The processed meat portion was reduced from 1.5 oz, presented in the original AHEI-2010, to 1 oz due to the strength of evidence that processed meat increases colorectal cancer risk; the World Cancer Research Fund (WCRF) Continuous Update Project identified a 16% increased risk of colorectal cancer per 50g increase in processed meat consumed per day (RR 1.16 [95% CI 1.08–1.26] following dose–response meta-analyses of cohort studies on consumption of processed meat and the risk of cancer ^[25]^ Processed meat comsumption limits are similar in France at a maximum of 25g per day ^[26][27]^.

^i^ Highest and lowest deciles based on intakes recorded for the UK arm of the Food4Me study (unpublished data).

^j^ Non-alcohol drinkers scored 100 points (modified from AHEI-2010 where non-alcohol drinkers (classified as <0.5 drinks/d) received a score of 2.5 out of 10). This component was modified to 100 points for both low and non-alcohol drinkers to discourage non-drinkers from introducing alcohol into their diets. This type of recommendation was considered inappropriate, especially due to the challenges related to alcoholism ^[28]^.
