# Supplementary material for: Effectiveness of Web-Based Personalized Nutrition Advice for Adults Using the eNutri Web App: Evidence From the EatWellUK Randomized Controlled Trial
Source: J Med Internet Res. 2022 Apr 25;24(4):e29088. doi: 10.2196/29088 (PMC9154737; doi:10.2196/29088)
Supplement: Multimedia Appendix 2 [file jmir_v24i4e29088_app2.docx]

**Multimedia Appendix 2**


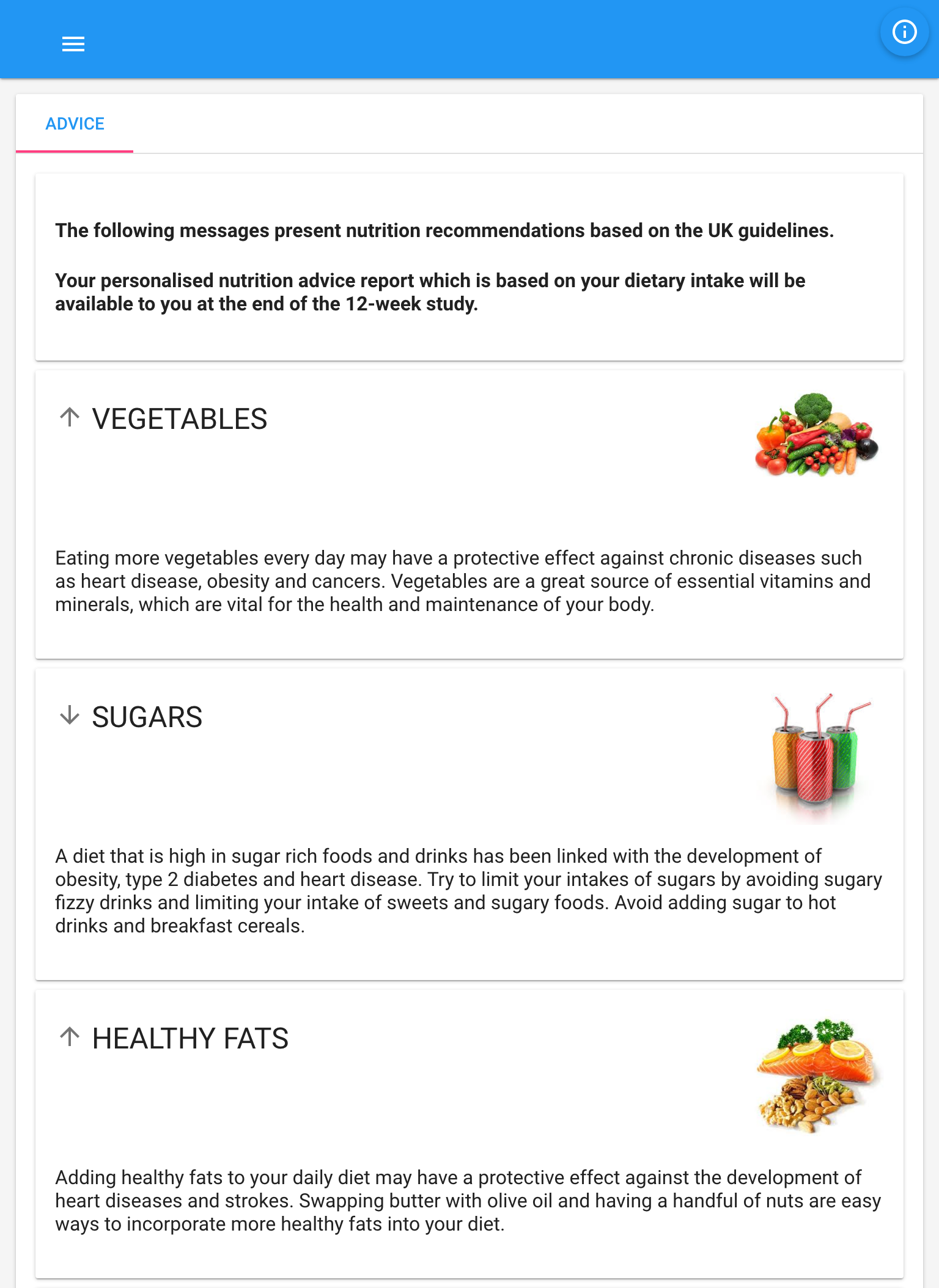


**Supplementary Figure 1. Nutrition advice received by the control group participants**


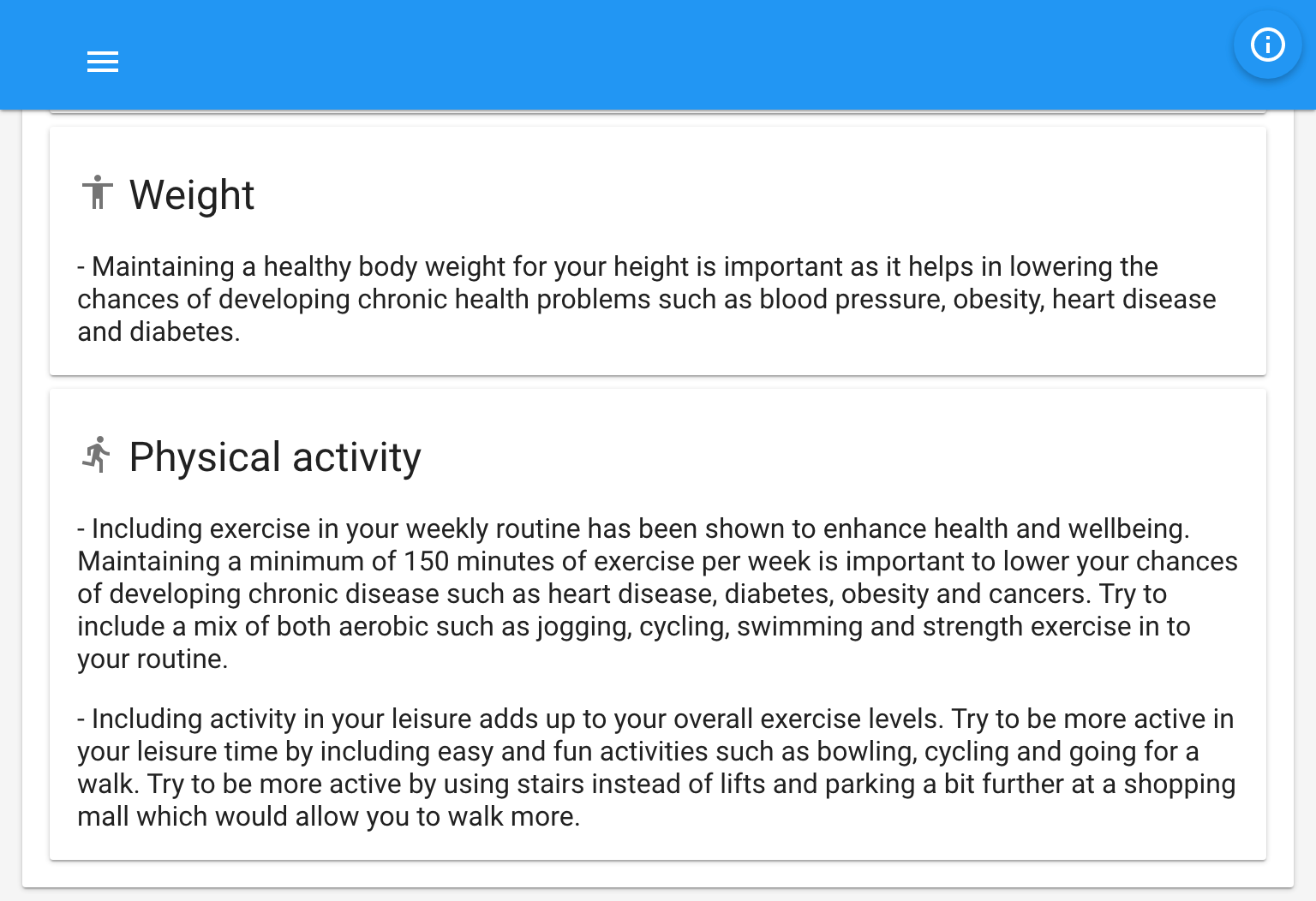


**Supplementary Figure 2. Weight and physical activity advice received by the control group participants**
